# Decreased Expression of *BNC1* and *BNC2* Is Associated with Genetic or Epigenetic Regulation in Hepatocellular Carcinoma

**DOI:** 10.3390/ijms17020153

**Published:** 2016-01-25

**Authors:** Yali Wu, Xiaolei Zhang, Yongzhen Liu, Fengmin Lu, Xiangmei Chen

**Affiliations:** Department of Microbiology & Infectious Disease Center, School of Basic Medical Science, Peking University Health Science Center, Beijing 100191, China; yaliwu@bjmu.edu.cn (Y.W.); xiaoleizhang8802@bjmu.edu.cn (X.Z.); luzernliu@bjmu.edu.cn (Y.L.); lu.fengmin@hsc.pku.edu.cn (F.L.)

**Keywords:** Basonuclin1, Basonuclin2, hepatocellular carcinoma, methylation, deletion

## Abstract

The aberrant expression of transcription factor Basonuclin (*BNC*) had been reported in different kinds of tumors. Here, we investigated the expression and methylation status of two Basonuclin homologs, *BNC1* and *BNC2* in hepatocellular carcinoma (HCC). We found that the expression levels of both *BNC1* and *BNC2* were down-regulated in HCC cell lines and primary HCC tissues. The frequency and intensity of *BNC1* promoter hypermethylation in tumor tissues was significantly higher than that in adjacent non-tumor tissues. 5-Aza-2’-Deoxycytidine treatment could significantly increase the *BNC1* expression in the methylated HCC cell lines, which implicated that epigenetic modification contributed to the down-regulation of *BNC1*. In addition, *BNC1* hypermethylation in tumor tissues was more likely to happen in female patients. No methylation of the *BNC2* promoter was found in HCC tumor tissues. However, a frequent deletion of the *BNC2* gene was observed, which indicated that the chromosomal loss of the *BNC2* gene might be one important reason for its lower expression level in HCC. Our results suggested that *BNC1* and *BNC2* were down-regulated in HCC which may provide new insight into the tumorigenesis of HCC.

## 1. Introduction

Hepatocellular carcinoma (HCC) is the second leading cause of cancer-related deaths worldwide and China alone accounts for 50% of the total number of deaths [1]. There is growing evidence that the aberrant expression of cellular genes in liver cells, due to genetic and/or epigenetic alterations, may contribute to the development of HCC [2,3]. Therefore, identifying new molecular carcinogenic events will help us to further understand the mechanisms of HCC and give guidance for the development of novel clinical treatment.

Basonuclin (*BNC*) is a cell-type-specific zinc finger protein which is quite conserved in evolution. Basonuclin 1 (*BNC1*) was firstly identified in cultured human epidermal keratinocytes and was highly expressed in skin keratinocytes and germ cells of the testis and ovary [4,5]. The human *BNC1* gene is located on chromosome 15 and possesses three separated pairs of zinc fingers, a nuclear localization signal and a serine strip [4,6]. It has been reported that *BNC1* may function as a transcription factor which could interact with a subset of genes that are transcribed by both RNA polymerase I (Pol I) and polymerase II (Pol II) and also has a role in maintaining ribosome production and regulating the expression of genes involved in cellular differentiation and proliferation [7,8]. Recently, another Basonuclin gene (*BNC2*) was reported [5], which had a common evolutionary origin with *BNC1*. Although the amino acid sequence of *BNC2* differs extensively from that of *BNC1*, *BNC2* possesses all of the characteristic features of *BNC1* described above, and has a wider distribution than *BNC1*. Since *BNC2* might co-localize with serine/arginine-rich splicing factor 2 (SRSF2/SC35), a splicing factor in keratinocytes, its function was considered likely to be related to mRNA splicing or other forms of mRNA processing [8].

Numerous lines of investigation have demonstrated that the aberrant expression of *BNC1* and *BNC2* contribute to tumor progression. *BNC1* has been shown to be silenced by promoter methylation in a wide variety of tumors, including pancreatic cancer, renal cell carcinoma, lung cancer, lymphocytic leukemia and the metastatic brain tumors originating from primary breast tumors [9,10,11,12,13]. Over-expression of *BNC1* in pancreatic cancer cell lines inhibited colony formation and cell proliferation *in vitro* [9]. Thus, *BNC1* was thought to be a potential tumor suppressor gene in these tumors. However, in breast cancer and squamous cell carcinoma of the head and neck [7,14], *BNC1* expression was elevated with increased invasive and metastatic capacity. In line with this, loss of *BNC1* expression resulted in a reduced epithelial-mesenchymal transition (EMT) phenotype, suggesting that the expression of *BNC1* would enhance the process of metastasis via EMT [13]. These findings suggested that *BNC1* might play diverse roles in tumor progression depending on cellular context and disease stage. Little is known about the expression and function of *BNC2* in tumor progression. According to reports, *BNC2* was associated with skin color variation and skin cancer risk [15]. Moreover, deletion of the *BNC2* gene and decreased expression of *BNC2* mRNA have been detected in Barrett’s esophagus tumor tissues. In esophageal adenocarcinoma cells, stable expression of *BNC2* caused the growth arrest of tumor cells [16], suggesting that *BNC2* might also be a tumor suppressor gene. To date, the roles of the *BNC1* and *BNC2* genes in hepatocarcinogenesis have not been addressed.

In this study, the expression levels and methylation statuses of *BNC1* and *BNC2* were investigated in primary HCC tissues and their corresponding adjacent non-tumor liver tissues, in order to investigate the potential roles of *BNC1* and *BNC2* genes in HCC.

## 2. Results

### 2.1. The Expression of Basonuclin 1 (BNC1) and Basonuclin 2 (BNC2) Genes Were Down-Regulated in Hepatocellular Carcinoma (HCC) Cell Lines and Primary HCC Tissues

**Figure 1:**
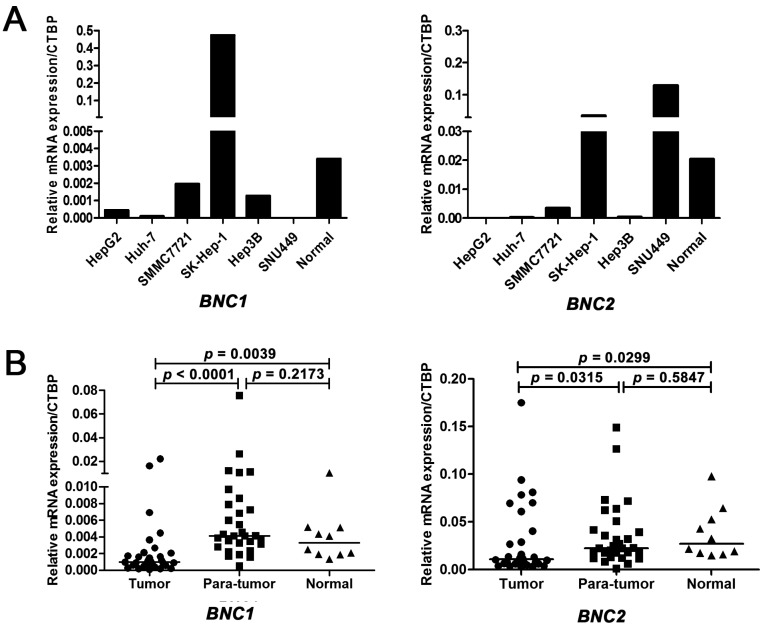
The mRNA expression levels of Basonuclin (*BNC*) genes in liver cancer cell lines and primary hepatocellular carcinoma (HCC) tissues detected by real-time RT-qPCR. (**A**) Expression of Basonuclin 1 (*BNC1*) and Basonuclin 2 (*BNC2*) in liver cancer cell lines; (**B**) Expression of *BNC1* and *BNC2* in 30 pairs of HCC tumor tissues and adjacent non-tumor tissues and 10 normal liver tissues. CTBP1: C-terminal binding protein 1.

Firstly, real-time RT-qPCR was used to detect the mRNA expression levels of *BNC1* and *BNC2* in five HCC cell lines (HepG2, Huh-7, SMMC7721, Hep3B, and SNU449) and one liver adenocarcinoma cell line (SK-Hep-1). As shown in Figure 1A, much lower mRNA expression levels of *BNC1* was observed in all five HCC cell lines than that in normal liver tissues. Similar to *BNC1*, *BNC2* mRNA expression levels were decreased in four HCC cell lines including HepG2, Huh-7, SMMC7721 and Hep3B cells. Both *BNC1* and *BNC2* mRNA expression levels were higher in the liver adenocarcinoma cell line SK-Hep-1 than in normal tissue. Next, we tested the expression levels of *BNC1* and *BNC2* in 30 pairs of matched tumor and their adjacent non-tumor tissues. Consistent with the results in HCC cell lines, both *BNC1* and *BNC2* mRNA expression levels in tumor tissues were statistically lower than that in corresponding non-tumor tissues (*p* < 0.0001 and *p* = 0.0315, respectively), as well as that in normal liver tissues (*p* = 0.0039 and *p* = 0.0299, respectively) (Figure 1B).

### 2.2. Chromosomal Loss of BNC1 and BNC2 Genes in Primary HCC Tumor Tissues

**Figure 2:**
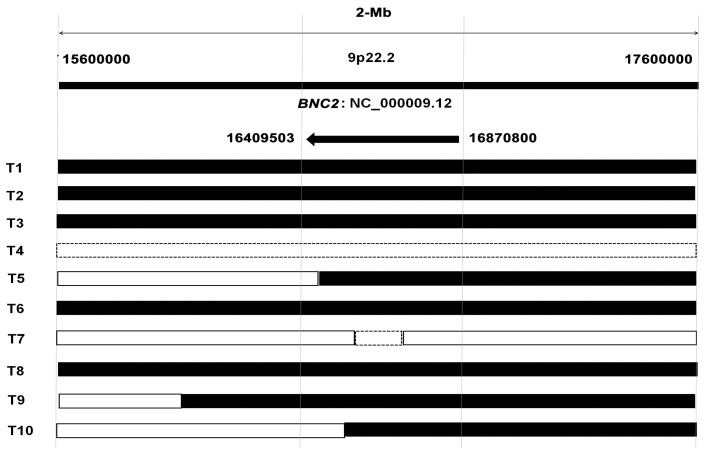
Chromosomal aberrance of *BNC2* gene in HCC tissues. “T” represents tumor tissue; Black lines represent Chromosome deletion; the dotted lines represent Chromosome amplification and real lines represent normal status. 2-Mb: 2-Megabase.

To detect the copy number aberrations of genes in HCC tissues, we have previously performed a 2-Megabase (2-Mb) array-based comparative genomic hybridization (aCGH) analysis in 25 pairs of HCC tissues and their corresponding non-tumor tissues [17]. The result revealed that the *BNC2* gene was deleted in eight HCC tissues, partially amplified in one case, and the whole gene was amplified in one case (Figure 2). Since partial amplification of the *BNC2* gene could also lead to expression silence, the rate of chromosomal loss in the *BNC2* gene was (9/25; 36%). As for the *BNC1* gene, the deletion rate was only 8% (2/25). These results indicate that the frequent loss of the *BNC2* gene might be an important reason for its low mRNA expression in HCC; while for the *BNC1* gene, mechanisms other than chromosome loss might lead to its down-regulation in HCC.

### 2.3. Promoter Methylation of BNC1 and BNC2 in HCC Cell Lines

**Figure 3:**
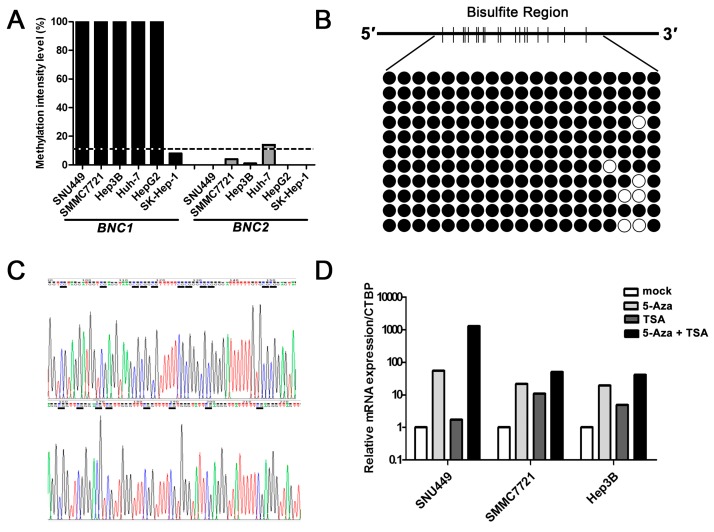
Promoter methylation status of *BNC1* and *BNC2* in liver cancer cell lines. (**A**) The methylation status of *BNC1* and *BNC2* genes in five HCC cell lines and liver adenocarcinoma cell line SK-Hep-1 detected by methylation-sensitive restriction enzymes-based quantitative PCR (MSRE-qPCR). The dotted line represents the 10% cut off value; (**B**) Bisulfite sequencing analysis of the *BNC1* CpG island in Hep3B cells. Short vertical bars represent CG sites; black circles represent methylated CG sites; white circles represent unmethylated CG sites; (**C**) A representative bisulfite sequencing chromatogram of *BNC1* CpG island in Hep3B cells. The green lines represent adenine “A”, blue lines represent cytosine “C”, black lines represent guanine “G” and red lines represent thymine “T”; (**D**) *BNC1* expression in three HCC cell lines after treatment with 5-Aza-2′-Deoxycytidine, trichostatin A (TSA) or both. The mock group was normalized as 1.

Promoter CpG island (CGI) hypermethylation is an important mechanism for the inactivation of tumor suppressor gene in HCC. To detect whether the promoter CGI region of *BNC1* or *BNC2* was hypermethylated in HCC, methylation-sensitive restriction enzymes-based quantitative PCR (MSRE-qPCR) was performed in five HCC cell lines and the liver adenocarcinoma cell line SK-Hep-1. With threshold value of methylation intensity set at 10% [18], a strong hypermethylation status of *BNC1* was detected in all five HCC cell lines (Figure 3A). A further bisulfite sequencing assay also confirmed the hypermethylation status of the *BNC1* promoter region in Hep3B cells (Figure 3B,C). No methylation was found in SK-Hep1 cells which showed higher expression of *BNC1* mRNA. Unlike *BNC1*, *BNC2* methylation was only found in Huh7 cells (Figure 3A).

To further test whether DNA methylation suppressed *BNC1* expression, we treated SNU449, SMMC7721 and Hep3B cells with DNA methyltransferase inhibitor 5-Aza-2′-Deoxycytidine, and analyzed the changes of *BNC1* expression by real-time RT-qPCR. As shown in Figure 3D, treatment with 5-Aza-2′-Deoxycytidine significantly enhanced *BNC1* expression in these cells. Additionally, treatment of the SMMC7721 and Hep3B cells with only the histone deacetylation inhibitor trichostatin A (TSA), or together with 5-Aza-2′-Deoxycytidine also resulted in reactivation of *BNC1* expression. These results implicated that epigenetic modification in the promoter CGI region might modulate *BNC1* expression in HCC cells.

### 2.4. Methylation Status of BNC1 in Primary HCC Tissues

**Figure 4:**
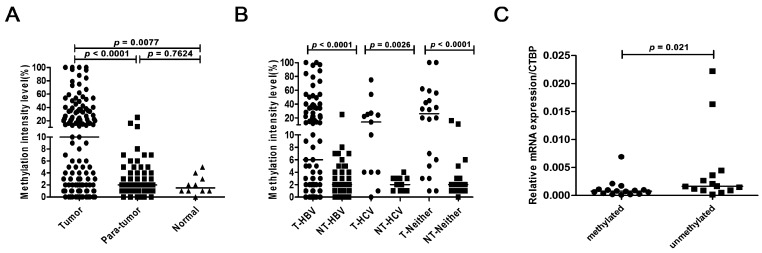
Methylation status of BNC1 in HCC tissues. (**A**) The methylation intensity of BNC1 in 127 pairs of HCC tumor tissues and adjacent non-tumor tissues and 10 normal liver tissues; (**B**) The methylation intensity of BNC1 in paired tumor tissues with different infection background. “T” represents tumor tissue; “NT” represents non-tumor tissue; “−HBV” represents with hepatitis B virus (HBV) infection; “−HCV” represents with hepatitis C virus (HCV) infection; “−Neither” represents without evidence of hepatitis viral infection; (**C**) The expression level of BNC1 mRNA in methylated HCC tissues (methylation intensity ≥ 10%) and unmethylated tissues (methylation intensity <10%).The horizontal black lines in the picture represent mean values.

Because of the higher frequency of the *BNC1* methylation detected in HCC cell lines, we then tested the methylation status of *BNC1* in tumor and adjacent non-tumor tissues derived from 127 primary HCC patients and 10 normal liver tissues. With threshold value of methylation intensity set at 10%, the high frequency of *BNC1* hypermethylation was found in tumor tissues (49.6%, 63/127), but not in the adjacent non-tumor tissues (3/127, 2.36%). No hypermethylation of *BNC1* was found in normal liver tissues. Moreover, the hypermethylation intensity in HCC tumor tissues was significantly higher than that in the adjacent non-tumor tissues (*p* < 0.0001) and normal liver tissues (*p* = 0.0077) (Figure 4A). Further stratified statistic analysis was conducted to test if the intensity of *BNC1* promoter hypermethylation was different in HCC patients with different etiology. It revealed no differences of *BNC1* promoter hypermethylation intensity in tumor tissues among patients with chronic hepatitis B virus (HBV) infection, with chronic hepatitis C virus (HCV) infection, and those without evidence of hepatitis viral infection. But the *BNC1* promoter hypermethylation intensity in tumor tissues with different infection backgrounds was higher than that in each group of corresponding non-tumor tissues (Figure 4B). In addition, we also detected the methylation status of *BNC2* in 16 pairs of tumor and adjacent non-tumor tissues (Figure S1). In line with the results in liver cancer cell lines, no methylation of the *BNC2* gene was identified in HCC tumor tissues.

To further demonstrate the correlation between methylation and *BNC1* expression, we divided the 30 tumor samples with *BNC1* expression tested into two groups according to *BNC1* methylation status [18], with the methylated group (methylation intensity ≥ 10%) and the unmethylated group (methylation intensity < 10%). Then we compared the *BNC1* expression in the two groups. As shown in Figure 4C, the expression level of the *BNC1* gene in the methylated group was significantly lower than that in the unmethylated group (*p* = 0.021), suggesting that the increase of *BNC1* methylation intensity in HCC might contribute to the down-regulation of *BNC1* expression.

### 2.5. Correlation between BNC1 Hypermethylation and Clinicopathological Features

To investigate the clinical significance of *BNC1* promoter CGI methylation, Chi-square test and Fisher’s exact test were used to analyze the relationship between *BNC1* methylation and patients’ clinicopathological variables. The number of patients for the association study ranged from 120 to 127, because of missing clinical data of some patients. The results showed that the hypermethylation of *BNC1* did not correlate to patients’ age, tumor size, tumor number, portal vein invasion and Barcelona Clinic Liver Cancer (BCLC) stage. But an increase of *BNC1* CGI hypermethylation happened more frequently in female patients in this cohort (*p* = 0.019) (Table 1). To understand the prognostic significance of aberrantly *BNC1* methylation in HCC, we also analyzed the correlation between *BNC1* methylation and patients’ overall post-surgery survival period. However, no prognostic significance of *BNC1* methylation was found in our current study (data not shown).

**Table 1:** Basonuclin 1 (*BNC1*) CpG island (CGI) hypermethylation and clinicopathological correlations in hepatocellularcarcinoma (HCC).

Feature		Hypermethylated (*n* = 63)	Unmethylated (*n* = 64)	*p*-Value
Gender	Male	37	50	0.019
	Female	26	14	
Age (years)	≥50	45	48	0.649
	<50	18	16	
AFP (μg/L)	≥400	31	24	0.180
	<400	31	39	
Tumor diameter	≥5cm	48	47	0.721
	<5cm	15	17	
Tumor number	1	50	45	0.240
	≥2	13	19	
Portal Vein Invasion	yes	7	15	0.072
	no	54	48	
BCLC stage	A	31	35	0.536
	B or C	32	29	

AFP: α Fetal Protein; BCLC: Barcelona Clinic Liver Cancer ; A: early stage; B: intermediate stage; C: advanced stage.

## 3. Discussion 

Basonuclin, a cell-type-specific zinc finger protein, is thought to be a transcription factor which maintains the proliferative capacity of keratinocytes and prevents their terminal differentiation [19]. However, the involvement of Basonuclin in the carcinogenesis of HCC remains unclear. In this study, we analyzed the expression and methylation statuses of two Basonuclin homologs, *BNC1* and *BNC2* in HCC.

Aberrant *BNC1* silence induced by promoter hypermethylation has been reported in several types of human tumors, including pancreatic cancer, renal cell carcinoma, lung cancer, lymphocytic leukemia and the metastatic brain tumors [9,10,11,12,13]. Consistent with these results, we found that *BNC1* was hypermethylated in HCC cell lines. Moreover, *BNC1* was down-regulated in mRNA level in HCC cells. The down-regulation of *BNC1* could be reversed by DNA methyltransferase inhibitor treatment, indicating that promoter methylation is one of the mechanisms for *BNC1* inactivation in HCC cell lines. By using a HCC patient cohort, we further demonstrated that *BNC1* was remarkably down-regulated in HCC tissues, which was largely contributed by DNA methylation of *BNC1*. In addition, we noticed that histone deacetylation inhibitor TSA also could increase the *BNC1* expression in HCC cell lines, suggesting that other mechanisms including the histone deacetylation modulation may lead to the reduced expression of *BNC1* in HCC. Unlike HCC cell lines, SK-Hep-1 cell was of endothelial origin which was derived from the ascitic fluid of a patient with adenocarcinoma of the liver [20]. In this study, we found that SK-Hep-1 had a relatively high level of *BNC1* expression. Concordantly, no methylation was found in *BNC* promoter region in SK-Hep-1 cells. These results further demonstrated that the methylation and expression patterns of *BNC1* in tumors appeared to be tissue and context-specific.

HBV or HCV infection is a major risk factor for the development of HCC in China. It has been reported that hepatitis B virus x protein (HBx) played an important role in DNA methylation by up-regulating DNA methyltransferases (DNMT) [21]. In this study, we compared the hypermethyaltion intensity of the *BNC1* promoter in HCC tissues with different infection backgrounds. We found that the hypermethylation intensity in tumor tissues was significantly higher than that in non-tumor tissues, whether with or without hepatitis virus infection. Thus, we concluded that the hypermethylation of *BNC1* might be a common mechanism in HCC tissues.

We also analyzed the clinical significance of the *BNC1* promoter in our current study. Unfortunately, no correlation between *BNC1* methylation and patients’ overall post-surgery survival period was found in our cohort. However, we observed a higher rate of *BNC1* methylation in female patients. Our previous studies have shown that the hypermethylation of *p16INK4a* [18] and suppressor of cytokine signaling 1 (SOCS1) [22] were more likely to happen in male gender patients. One possible reason for the different distribution of gene methylation between males and females might be the influence of specific sex hormones. Further studies should be performed in a larger cohort to confirm the potential correlation between *BNC1* methylation and the patient’s gender. In addition, previous studies have shown that over-expression of *BNC1* in pancreatic cancer cell lines could inhibit colony formation and cell proliferation, suggesting that *BNC1* might function as a tumor suppressor gene and its down-regulation in pancreatic cancer might contribute to tumor development and progression. Since *BNC1* was silenced by hypermethylation in HCC, functional assays should be performed *in vitro* and *in vivo* to investigate whether *BNC1* also exhibits anti-tumor function in the development of HCC in the future.

The *BNC2* gene is located on human chromosome 9. Although the amino acid sequence of *BNC2* differs extensively from that of *BNC1*, the two proteins share some essential features. *BNC2* was found in virtually every cell type and the extreme conservation of the *BNC2* amino acid sequence across vertebrates suggested that *BNC2* might play an important role, presumably as a regulatory protein of DNA transcription [6]. However, little is known about the roles of *BNC2* in tumorigenesis. In this study, we found that the expression of *BNC2* was also down-regulated in HCC cell lines and HCC tissues. Unlike *BNC1*, the promoter region of *BNC2* was seldom hypermethylated in both liver cancer cell lines and HCC tissues. But the *BNC2* gene was frequently deleted in HCC tissues, indicating that chromosomal loss of the *BNC2* gene might be one important reason for its low mRNA expression in HCC.

In summary, this study has shown for the first time that both *BNC1* and *BNC2* are down-regulated in HCC tissues and HCC cell lines through epigenetic silencing or gene deletion. The identification of the epigenetic inactivation of *BNC1* gene may provide insight into HCC tumorigenesis and provide a basis for developing novel biomarkers for HCC prognosis and therapy.

## 4. Materials and Methods

### 4.1. Tissue Samples and HCC Cell Lines

With the ethics committee approval (1 January 2013, IRB00001052-12088) and patients’ informed consent, 127 pairs of primary HCC tissues and the adjacent non-tumor tissues from Henan Oncology Hospital (Zhengzhou, China) (from 2010 to 2012) were enrolled in our study. All the patients were diagnosed with HCC and confirmed by liver biopsy. The clinicopathological characteristics of the HCC patients are shown in Table 2. Normal liver tissues (*n* = 10) were obtained from liver donors in the same hospital.

Human liver cancer cell lines (HepG2, Huh-7, SK-Hep-1, Hep3B and SNU-449) were purchased from ATCC (Manassas, VA, USA) and Cell Resources Center of Peking Union Medical College (Beijing, China) (SMMC7721). HepG2, Huh-7, SK-Hep-1 and Hep3B were cultured in DMEM (Gibco, Carlsbad, CA, USA); SNU-449 and SMMC7721 were maintained in RPMI 1640 (Gibco).

### 4.2. Gene Expression Analysis

Real-Time RT-qPCR was performed to measure the expression level of *BNC1* and *BNC2* gene using a Roche light cycle 480 sequence detection system (Roche, Mannheim, Germany). Primers used for detection of *BNC1* expression were forward primer: 5′-GGCCGAGGCTATCAGCTGTACT-3′ and reverse primer: 5′-GCCTGGGTCCCATAGAGCAT-3′. Primers used for detection of *BNC2* expression were forward primer: 5′-TGACAACCAGCATGCCGAGA-3′ and reverse primer: 5′-ATCAACCCCACAACATGGGA-3′. C-terminal binding protein 1 (*CTBP1*) was included as a house keeping gene control to normalize expression levels. Primers used for detection of *CTBP1* expression were forward primer: 5′-TTCACCGTCAAGCAGATGAGAC-3′ and reverse primer: 5′-CTGGCTAAAGCTGAAGGGTTCC-3′. Each experiment was done in duplicate and the expression levels of *BNC1* and *BNC2* were determined by the comparative *C*t method (2^−Δ*C*t^) after normalization to the *CTBP1* [18].

**Table 2:** Clinicopathological parameters of 127 patients with HCC.

Clinicopathological Parameters Variables		Cases n (%) *n* = 127
Age	Median (Range)	57 (11–80)
Gender	Male	87 (68.50)
	Female	40 (31.50)
Liver cirrhosis	Yes	115 (90.55)
	No	10 (7.87)
	N/A	2 (1.58)
Portal vein tumor thrombosis	Present	22 (17.32)
	Absent	102 (80.32)
	N/A	3 (2.36)
Tumor size	≥5 cm	96 (75.59)
	<5 cm	31 (24.41)
Infection background	HBV	94 (70.02)
	HCV	13 (10.24)
	Without HBV or HCV	20 (15.75)
Tumor encapsulation	Complete	104 (81.89)
	Uncomplete	16 (12.60)
BCLC stage	N/A	7 (5.51)
	A	66(51.97)
	B	17(13.39)
	C	44(34.64)
	D	0

N/A: Not available; HBV: hepatitis B virus; HCV: hepatitis C virus; BCLC: Barcelona Clinic Liver Cancer; A: early stage; B: intermediate stage; C: advanced stage; D: terminal stage.

### 4.3. High-Density Oligonucleotide aCGH Analysis

Chromosome aberration was comprehensively analyzed by using the Aiglent Human Genome 244K CGH microarray (Shanghai Bio corporation, Shanghai, China) in 25 pairs of HCC tissues and adjacent non-tumor tissues [17].

### 4.4. CpG Island (CGI) Methylation Assay

The quantificational methylated DNA analysis was performed by methylation-sensitive restriction enzymes-based quantitative PCR (MSRE-qPCR) as described previously [18].The CGI region of *BNC1* gene starts from −462 and ends at +1488 nt predicted using online program CpG Island Searcher. The chosen target region for quantificational methylation analysis of *BNC1* CGI region covered 75 bp in the CGI region which contains 4 HhaI cutting sites (from +166 to +241 of transcriptionstart site) (Figure S2A). The primer sequences of *BNC1*were as follows: forward: 5′-AGCGGTCGCAGGATGGCCGAGGT-3′, reverse: 5′-CCGCCTGCTCCGCGCACAGAGAATC-3′. The CGI region of *BNC2* gene starts from −1234 and ends at +663 nt. The chosen target region for quantificational methylation analysis of *BNC2* CGI region covered 114 bp which also contains 4 HhaI cutting sites (from −96 to +18 of transcriptionstart site) (Figure S2B). The primer sequences of *BNC2* were as follows: forward: 5′-GCGACCCCGGCGCGAGTGTAAATCA-3′, reverse: 5′-CGAGTTCCTCCGGGCCTCCGCTCTC-3′. In accordance to our previous reports, 10% was used as a cut-off value to differentiate hypermethylation and unmethylation [18].

### 4.5. Bisulfite Sequencing

Genomic DNA extracted from hepatocellular cell lines was treated with sodium bisulfite as reported previously [18,23]. 2 μg DNA was dissolved into 50 μL distilled water and 2 M NaOH was added at 37 °C for 10 min. To this, 10 mM hydroquinone and 3 M sodium bisulfite was added and incubated at 50 °C for 16 h. Then the DNA was purified with the Wizard DNA Clean-Up System (Promega, Madison, WI, USA). Next, the DNA was precipitated with ammoniumacetate and ethanol, washed with 70% ethanol, the pellet was dried, and then resuspended in 20 μL of distilled water. The bisulfite-modified DNA was amplified by PCR using the *BNC1* forward primer: 5′-GGTCGTAGGATGGTCGAGGTAAG-3′ and reverse primer: 5′-AACTAACCCTCGAAAAATAACCCTC-3′. Then the PCR product was purified and sequenced. The sequences were analyzed using BIOEDIT.

### 4.6. 5-Aza-2′-deoxycytidine Treatment in HCC Cell Lines

Human HCC cell lines SNU449, SMMC7721 and Hep3B were seeded in 6-well plates at a concentration of 2 × 10^5^ to 2.5 × 10^5^ cells per well. After 24 h, the medium was changed and cells were treated with 2 μM of 5-Aza-2′-deoxycytidine for 3 days and/or 500 nM of trichostatin A for the last 24 h. Then cells were collected for RNA extraction.

### 4.7. Statistical Analyses

Student’s *t*-test was used to compare the difference between two groups. Chi-square test or Fisher’s exact test was used to analyze the relationship between *BNC1* CGI methylation status and the patients’ clinicopathological features. Log-rank test was used for prognostic significance analysis. All statistical analyses were performed using the Statistical Analysis System SPSS Statistics 17.0 (SPSS, Inc., Chicago, IL, USA). In all cases, *p* < 0.05 indicated that the difference was statistically significant.

## 5. Conclusions

Our study indicated that both *BNC1* and *BNC2* were down-regulated in HCC. The identification of the epigenetically inactivated *BNC1* gene may provide insight into HCC tumorigenesis and provide a basis for developing novel biomarkers for prognosis and therapy.

## Supplementary Materials

Supplementary materials can be found at http://www.mdpi.com/1422-0067/17/2/153/s1.

## Abbreviations

BNC1: Basonuclin1, BNC2: Basonuclin2, HCC: Hepatocellular carcinoma MSRE-qPCR: Methylation-sensitive restriction enzymes-based quantitative PCR, CGI: CpG island, TSA: trichostatin A.
