# Supplementary material for: Decreased Expression of BNC1 and BNC2 Is Associated with Genetic or Epigenetic Regulation in Hepatocellular Carcinoma
Source: Int J Mol Sci. 2016 Jan 25;17(2):153. doi: 10.3390/ijms17020153 (PMC4783887; doi:10.3390/ijms17020153)
Supplement: Supplementary file 1 [file ijms-17-00153-s001.pdf]

Int. J. Mol. Sci. **2016**, *17*, 153; doi:10.3390/ijms17020153

Supplementary Materials: Decreased Expression of *BNC1* and *BNC2* Is Associated with the Genetic or Epigenetic Regulation in Hepatocellular Carcinoma

Yali Wu, Xiaolei Zhang, Yongzhen Liu, Fengmin Lu and Xiangmei Chen

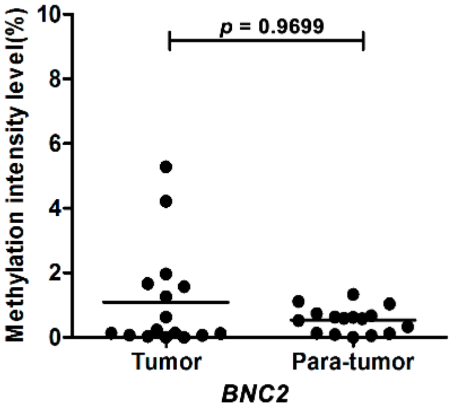

Figure S1. The methylation intensity of *BNC2* in 16 paired primary HCC tissues and their corresponding adjacent non-tumor liver tissues.

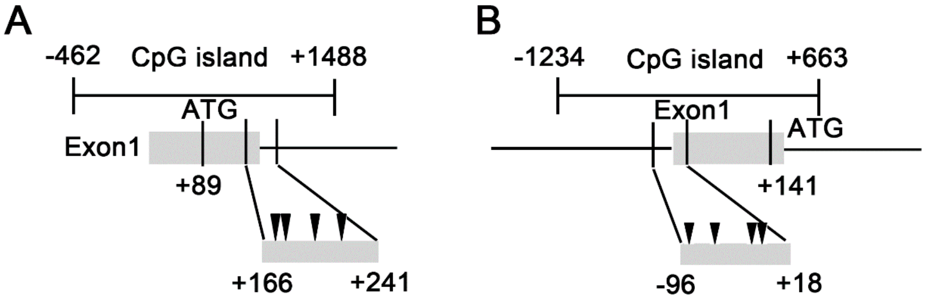

Figure S2. Schematic representation of the detected CGI loci of the (**A**) *BNC1* and (**B**) *BNC2* genes. The transcriptional start site for gene is defined as +1. Shaded boxes are exons of *BNC1* and *BNC2*. "▼" represents the restriction enzyme cutting site.
